# Hydrogen Separation Performance of UiO-66-NH_2_ Membranes Grown via Liquid-Phase Epitaxy Layer-by-Layer Deposition and One-Pot Synthesis

**DOI:** 10.3390/membranes11100735

**Published:** 2021-09-27

**Authors:** Alessandro Micero, Tawheed Hashem, Hartmut Gliemann, Aline Léon

**Affiliations:** 1 European Institute for Energy Research (EIFER), Emmy-Noether-Strasse 11, 76131 Karlsruhe, Germany; micero@eifer.org; 2 Institute of Functional Interfaces (IFG), Karlsruhe Institute of Technology (KIT), Hermann-von-Helmholtz-Platz 1, 76344 Eggenstein-Leopoldshafen, Germany; tawheed.hashem@kit.edu (T.H.); hartmut.gliemann@kit.edu (H.G.)

**Keywords:** hydrogen purification, liquid-phase epitaxy, layer-by-layer, UiO-66-NH_2_, SURMOF, membrane-based gas separation

## Abstract

The quality assurance of hydrogen fuel for mobile applications is assessed by the guidelines and directives given in the European and international standards. However, the presence of impurities in the hydrogen fuel, in particular nitrogen, water, and oxygen, is experienced in several refueling stations. Within this work, metal-organic framework (MOF)-based membranes are investigated as a fine-purification stage of the hydrogen fuel. Three H_2_/N_2_ concentrations have been used to analyze the separation factor of UiO-66-NH_2_ membranes prepared using the layer-by-layer (LBL) and the one-pot (OP) synthesis methods. It is shown that the separation factor for an equimolar ratio is 14.4% higher for the LBL sample compared to the OP membrane, suggesting a higher orientation and continuity of the LBL surface-supported metal-organic framework (SURMOF). Using an equimolar ratio of H_2_/N_2_, it is shown that selective separation of hydrogen over nitrogen occurs with a separation factor of 3.02 and 2.64 for the SURMOF and MOF membrane, respectively. To the best of our knowledge, this is the highest reported performance for a single-phase UiO-66-NH_2_ membrane. For higher hydrogen concentrations, the separation factor decreases due to reduced interactions between pore walls and N_2_ molecules.

## 1. Introduction

For sustainable mobility, the European Commission considers that hydrogen will account by 2050 for 32% of the total fuel mix in the European transport sector [1]. To achieve these targets, the employment of fuel cell light- and heavy-duty vehicles must be expanded as well as the necessary infrastructure, in particular the network of hydrogen refueling stations (HRSs). As of 31 March 2021, 445 HRSs have been installed worldwide, mainly in Germany (90) and Japan (134) [2,3].

In order to extend the performance and lifetime of the PEM fuel cell, the hydrogen fuel delivered at the nozzle must satisfy the Directive on Alternative Fuels Infrastructure (DAFI, Directive 2014/94/EU) and European (EN 17124:2018) as well as international (ISO 14687:2019) standards [4]. Indeed, the potential is high that some impurities in the level down to nmol/mol can degrade the fuel cell catalyst. In addition, between the production site (being off-site or on-site) and the nozzle, there is a long way where impurities can contaminate the hydrogen fuel during the production process, during transportation to the hydrogen station, or during the refilling process, including compression. That’s why, to ensure the quality of gaseous hydrogen fuel at the nozzle, four normative references, ISO-14687:2019, 19880-1, 19880-8:2019, and 21087, provide guidelines and directives to achieve the quality assurance of hydrogen in mobility applications.

The presence of impurities in the hydrogen stream is highly related to the hydrogen production methods. However, all impurities have to be currently measured regardless of on-site or off-site production. Hydrogen quality is generally determined either by off-site sampling or on-site monitoring using specific analyzers. The purity of the hydrogen fuel is determined by analyzing the concentrations of 14 contaminants (10 reactive gases, three inert gases, and particles) down to ppb level for some of them. As a result, the quality of hydrogen produced by centralized or decentralized (with on-site electrolyzers) systems requires certification before delivery to customers [5,6]. However, a recent four-year-long study about hydrogen quality has demonstrated that 29% of the samples collected from 28 different European HRSs violated the fuel quality limits and nitrogen was among the main contaminants. A survey conducted on more than 200 analyses at US HRSs revealed as well the presence of nitrogen as main impurity [7]. Consequently, fine-purification techniques are of interest to remove the remaining impurities in the hydrogen stream and provide the requested fuel quality assurance.

Separation and purification of gases in large-scale applications widely use pressure swing adsorption and cryogenic separation, while palladium membranes are frequently deployed in small-to-medium-scale applications [8]. An alternative to these existing technologies are membranes, which offer the potential to reduce energy consumption, [9,10] to simplify the operation, [11,12] and to be operated in multifunctional membrane reactors [13,14,15,16,17]. During the past decades, polymeric membranes, palladium-based membranes, [18,19,20,21,22] zeolite-based thin films, [23] and MOF-based membranes were investigated for separation processes, such as hydrogen purification, dewatering, CO_2_ removal from flue gases, olefin-paraffin separation, desulfurization, etc. [11,15,24].

Within this framework, this study aims to investigate the potential of MOF-based membranes to separate nitrogen impurities from hydrogen gas. MOFs are porous solid-state materials that consist of organic linker molecules (ligands) and metal (-containing) ions with tunable pores and a broad porosity range, [25] which can be prepared using various synthetic processes [26]. Among the 103,951 MOF structures determined so far, [27] it has been shown that MOFs based on tetravalent metal ions and carboxylate ligands, such as the zirconium-based UiO (Universitetet i Oslo) family, have extraordinary stability [23]. UiO-66, the most prominent member of this class, is a metal-organic framework made up of [Zr_6_O_4_(OH)_4_] clusters with 1,4-benzodicarboxylic acid linkers. It possesses centric octahedral cages linked with eight-corner tetrahedral cages through triangular windows of about 0.6 nm [28]. Further, grafting polar functional groups onto MOF organic ligands enhances gas separation [29], and, for example, this kind of functionalization has been shown to increase UiO-66 adsorption selectivity for CO_2_ over CH_4_ [30,31,32]. In this work, the amine-functionalized version, UiO-66-NH_2_, was selected with 2-amino-1,4-benzenedicarboxylic acid (NH_2_–BDC) linker because of its molecular sieving properties [30]. Although pore functionality and size of UiO-66-NH_2_ are different, the fcu topology is the same as the original MOF, preserving UiO-66′s excellent thermal and chemical stabilities [33,34]. In fact, the thermal stability of UiO-66-NH_2_ powder up to circa 450 °C has been determined via thermogravimetric analysis [34]. A comparison of the chemical structures of UiO-66 and UiO-66-NH_2_ can be found in the article by Kandiah et al. [33] UiO-66-NH_2_ synthesis using a liquid-phase epitaxial layer-by-layer (LPE–LBL) procedure to generate SURMOF films was accomplished using pre-synthesized Zr_6_O_4_(OH)_4_(OMc)_12_ SBUs (secondary building units) instead of the typical metal source, ZrCl_4_ [35]. Following that, synthesis parameters were modulated to improve crystallinity, resistance against hot water, high temperatures, and pH values between 2 and 10 [35].

In contrast to the established solvothermal method used for MOF production, the LPE–LBL synthesis introduced by Wöll and coworkers [36] was employed in this work. This process sequentially constructs SURMOF on a functionalized substrate at low temperatures with precise kinetic control [35]. First, a self-assembled monolayer (SAM) is generated on the support to serve as a suitable nucleation site for subsequent SURMOF growth. Depending on the materials used, chemical activation of the surface can be accomplished in a variety of ways, including UV irradiation, oxygen plasma treatment, or reaction with specific silanes (for oxidic interfaces, glass, etc.) or thiols (for metallic surfaces, such as Au, Ag, etc.). The SAM-functionalized substrate is then sequentially (1) dipped into a solution containing metal precursor, followed by (2) a rinsing stage, an immersion (3) into a solution with an organic linker, and a final (4) rinse. This process is repeated for a desired number of cycles and can be replaced by spin-coating [37] or spraying [38] techniques. The resulting SURMOF exhibits remarkable crystallinity, surface continuity, and orientation, [39] making it a promising candidate for gas separation [35]. In addition, UiO-66-NH_2_ SURMOF synthesized via a low-temperature LPE–LBL method shows outstanding resistance against hot water and high temperature [35].

In this work, UiO-66-NH_2_ films were grown on gold-coated alpha-alumina (α-Al_2_O_3_) using LPE–LBL (U-LBL) and one-pot synthesis (U-OP) procedures as described by Hashem et al. [34,35]. Prepared samples have been employed to determine their hydrogen permeance and their performance in hydrogen-nitrogen gas separation. Hydrogen was mixed with different concentrations of nitrogen to simulate conditions that can occur in an HRS. The gas separation performance of samples was evaluated using the Wicke–Kallenbach method. By that, the characterization of the membranes is first presented in terms of crystalline structure, chemical composition, and morphology, followed by the pressure-dependent permeation tests.

## 2. Materials and Methods

### 2.1. Materials

Porous α-Al_2_O_3_ disks (*h* = 1 mm, *d* = 13 mm, *ɛ* = 0.4–0.55; Fraunhofer IKTS) were used as substrate. These are special composite microfiltration membranes, consisting of a smooth layer of α-Al_2_O_3_ (*d_50_* = 70 nm) on the top of an α-Al_2_O_3_ support (*d_50_* = 2.5 μm).

Organic linker and metal ion sources were obtained from Alfa Aesar (Kandel, Germany) as 2-aminoterephthalic acid, C_8_H_7_NO_4_ (99%), and zirconium(IV) chloride, ZrCl_4_ (99.5%), respectively. For the functionalization, an 11-mercapto-1-undecanol (MUD) solution was purchased from Sigma-Aldrich (Darmstadt, Germany). The used solvents were hydrogen chloride (HCl), provided by VWR Chemicals (Radnor, PA, USA), and *N,N*-Dimethylformamide (DMF), supplied by Merck (Darmstadt, Germany). DMF was employed not only as a solvent for both linker and metal ion compounds but also for washing the synthesized samples. All chemicals and reagents were used as received without further purification.

### 2.2. Synthesis Procedures

The synthesis procedure of the membranes consists of functionalizing the surface of the α-Al_2_O_3_ substrate followed by the deposition of the UiO-66-NH_2_ either by layer-by-layer (U-LBL) or one-pot synthesis (U-OP) technique. A detailed description of the different processes is given in the following sections.

#### 2.2.1. Surface Functionalization Process

For all samples, a titanium layer (2–3 nm) was first sputtered on the α-Al_2_O_3_ surface, then a gold layer of about 70 nm was applied via physical vapor deposition. The metal coating was carried out in a self-made evaporation apparatus. In the first step, the Al_2_O_3_ substrates were tempered at 340 °C for 4 h at 10^−8^ mbar, cooled to room temperature (RT), and then first coated with 2 nm titanium and with 50 nm gold subsequently. The evaporation of the metals was carried out by indirect heating in a tungsten container. The growth rate was set to 0.5 nm/s for Ti and 20 nm/s for Au. The average pressure during coating was 10^−7^ mbar.

The intermediate titanium layer was used to improve the adhesion of the gold layer on the supporting substrate. It has been proved that the gold layer does not cause any pore blockage nor prevent diffusion of gases through the support. Instead, the gold coating improves the anchoring of SURMOF on its surface, increases homogeneity, and better defines the orientation of deposited crystals [40].

For functionalizing the substrate, the Au-coated α-Al_2_O_3_ was left immersed in a 1 mmol/L ethanolic MUD solution for 24 h in the dark at room temperature. This generated a self-assembled monolayer (SAM), characterized by the presence of –OH functional groups, which improves the attachment of the SURMOF. These functionalized supports were then washed with absolute ethanol and dried in a flow of pure nitrogen for immediate use in the synthesis of UiO-66-NH_2_.

#### 2.2.2. Synthesis of UiO-66-NH_2_ on α-Al_2_O_3_

##### UiO-66-NH_2_ Layer-by-Layer Synthesis Procedure (U-LBL)

**Figure 1:**
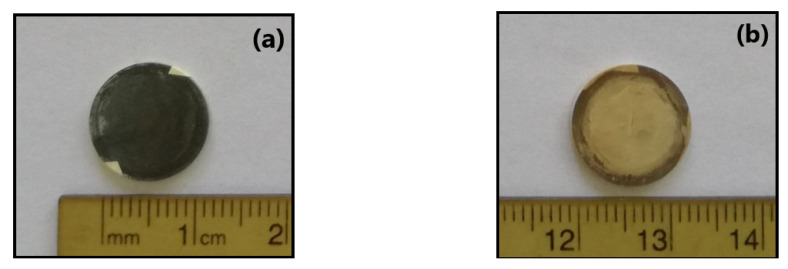
Pictures of the top surface of (**a**) U-LBL and (**b**) U-OP.

UiO-66-NH_2_ SURMOF was grown on the functionalized substrate through a manual dip-coating LPE–LBL process. Concentrations and synthesis conditions described in the following were adapted from the study by Hashem et al. [35]. Two solutions were prepared: the metal ion solution included 90 mg ZrCl_4_ dissolved in a mixture of 10 mL DMF and 2 mL (37 wt%) HCl, and the organic linker solution contained 150 mg 2-aminoterephthalic acid dissolved in 10 mL DMF. The temperature of the two solutions was brought to 70 °C and maintained at this value for all the following steps. First, the functionalized gold-coated substrate was (1) immersed in the ZrCl_4_ solution while stirring at 500 rpm for 1.5 h and (2) rinsed with DMF for 5 min. This enabled metal ions to form a coordinative bond with carboxylic functional groups grown on the substrate’s surface. Then, the support was (3) added to the ligand solution with continuous stirring at 500 rpm for 2 h and (4) washed with DMF for 5 min. This four-step dipping cycle was repeated 30 times to create as many UiO-66-NH_2_ layers on the functionalized Au-coated substrate. Thereafter, the prepared membranes were rinsed six times with ethanol and left in pure ethanol at room temperature for 24 h before being dried overnight in air. This sample is denoted as *U-LBL* and is depicted in Figure 1a, where the black color of the tarnished gold coating is evident. For comparison, a sister membrane, labeled *U-LBL-d*, was synthesized via the same method but on uncoated α-Al_2_O_3_ support.

##### UiO-66-NH_2_ One-Pot Synthesis Procedure (U-OP)

A one-pot synthesis procedure was developed to simplify the steps and the concentrations, as well as the synthesis conditions, adapted from the article by Hashem et al. [34]. For this synthesis, the metal ion solution was prepared with 125.8 mg ZrCl_4_ dissolved in a mixture of 5 mL DMF and 1 mL (37 wt%) HCl, and the organic linker solution with 135.8 mg 2-aminoterephthalic acid dissolved in 5 mL DMF. The functionalized α-Al_2_O_3_ surface support was first immersed in DMF while stirring for 5 min, then ZrCl_4_ solution was added with an increased stirring speed at 700 rpm for 10 min to enable metal ions to interact with carboxylic functional groups grown on the surface of the substrate. Thereafter, the ligand solution was added to the mixture, while the temperature was increased to 80 °C with continuous stirring. The color of the mixture rapidly changed from transparent to milky white. The prepared membranes were carefully taken out from the solution, rinsed several times with ethanol, and left in pure ethanol at room temperature for 24 h before being dried. The sample is labeled as *U-OP* and is illustrated in Figure 1b, where a typical yellow hue attributed to the linker present in the UiO-66-NH_2_ framework is noticeable [33,41,42]. For comparison, a sister membrane, denoted as *U-OP-d*, was synthesized via the same method but on uncoated α-Al_2_O_3_ support.

### 2.3. Sample Characterization

The membranes were characterized using X-ray diffraction (XRD), environmental scanning electron microscopy (ESEM), and attenuated total reflectance–Fourier transform infrared spectroscopy (ATR–FTIR) to assess their crystallinity, morphology, and chemical composition, respectively.

XRD symmetrical reflection measurements were conducted using a Bruker D8 Advance. The diffractometer is equipped with a 0.15419 nm Cu-Kα_1,2_ radiation, a variable divergence slit for the incident beam, and a silicon strip detector (Lynxeye) in *2θ–θ* geometry. The diffraction patterns were recorded over an angular range of 5.5–20°, with an increment of 0.02° and 1 s per step for all samples.

ESEM imaging was performed using FEI/Philips XL30 FEG (FEI company, Hillsboro, OR, USA). Membranes were coated with a thin conductive Au/Pd-film (ca. 5 nm thickness) to avoid the charging effect and increase the sample conductivity. The membranes were probed using acceleration voltages between 5 and 20 kV in a high-vacuum environment (5–10·10^–5^ bar).

ATR-FTIR measurements were performed using a Tensor 27 spectrometer (Bruker, Billerica, MA, USA), equipped with a Bruker Optics Platinum^®^ ATR assembly (diamond crystal with one reflection) and a room temperature deuterated triglycine sulfate detector. All spectra were recorded at room temperature from 4000 to 400 cm^−1^, with a resolution of 4 cm^−1^ under air.

### 2.4. Permeation Tests

After characterization, a permeation test was performed to evaluate the surface continuity of the membranes. The samples were inserted between two chambers of a gas-tight stainless-steel membrane module, each with an inlet and an outlet. Hydrogen was used as feed gas with a flow rate of 150 mL·min^−1^ and a pressure range from 1.12 to 1.42 bar. Argon was employed as sweep gas with a flow rate of 150 mL·min^−1^ and 1.1 bar as initial pressure. The permeate and retentate flow rates were determined by a Definer 220 flowmeter (MesaLabs, Lakewood, CO, USA) and a bubble flowmeter, respectively. The setup was used at room temperature since the chemical gradient potential was supposed to be the only driving force present. This was represented, to a first approximation, by the partial pressure gradient of the gases between the chambers [40]. The permeance measurements were performed once equilibrium was reached within the chambers.

### 2.5. Gas Separation Experiments

**Figure 2:**
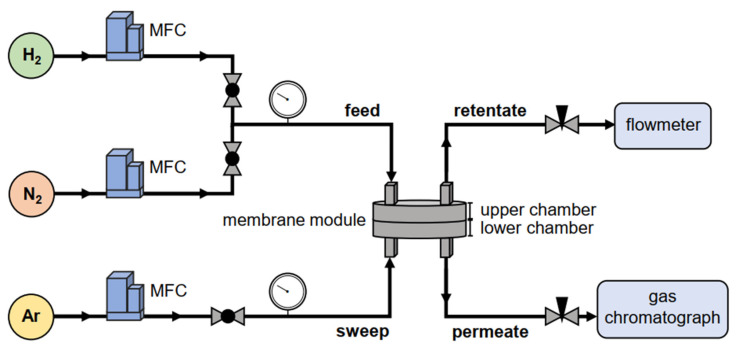
Scheme of the Wicke–Kallenbach apparatus used in gas separation experiments. It consists of two inlets (feed and sweep flows), two outlets (retentate and permeate flows), three mass flow controllers (MFCs), two needle valves, a flowmeter, and a gas chromatograph. The sample is placed between the upper and lower chambers of the membrane module.

A typical Wicke–Kallenbach diffusion cell (Figure 2) was used at room temperature for gas separation experiments. The sample was placed between the chambers of the membrane module, which is the same used for the permeation test. The binary-gas mixture (composed of hydrogen and nitrogen) was fed into the inlet of the top chamber, while the sweep gas (argon) entered the lower inlet. The retentate flow exited the upper outlet, and its flow rate was measured by a bubble flowmeter. The permeate, namely the purified mixture, exited the lower outlet, and its composition was analyzed via gas chromatography, using argon as the carrier gas. The flow rates of inlet gases were governed by mass flow controllers. Needle valves on both permeate and retentate sides were used to guarantee equilibrium, i.e., no pressure gradient across the membrane module to exclude forced flow as a transport mechanism [39,43]. For the entire duration of experiments, a high-sensitivity digital manometer checked that chamber pressure drop was kept below ±5 mbar while the absolute pressure was maintained in the range of 1.14–1.18 bar (equal on both chambers). A preliminary stabilization phase of about 20 min preceded each measurement to reach a steady-state condition before the beginning of the test.

The flow rates of the feed and sweep gases were both set at a constant flow rate of 50 mL·min^−1^ to ensure a balance in the module. The feed gas had different concentration ratios: equimolar (1:1) taken as reference and hydrogen-rich (5:1 and 10:1) as a case study. The gas chromatograph used for gas analysis is a 7890B (Agilent Technologies, Santa Clara, CA, USA) equipped with HP-Plot Q and HP-Molsieve columns (Agilent J&W, Santa Clara, CA, USA). The calibration of MFCs and GC systems was performed using calibrated flow and gases.

## 3. Results

### 3.1. Sample Characterization

**Figure 3:**
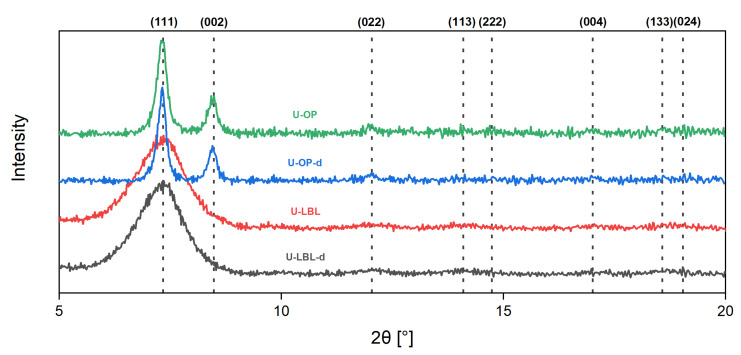
Normalized diffractograms of the four UiO-66-NH_2_ membranes. The plots of U-LBL and U-LBL-d have been recently published elsewhere [35].

Figure 3 displays the XRD patterns of the synthesized membranes. The diffractograms were normalized to the highest peak intensity, which is the one corresponding to the (111) plane. The patterns of the LBL samples were extracted from the article by Hashem et al. [35]. The peaks in the range *2θ* = 5.5°–20° correspond to the following planes: (111) to 7.40°, (002) to 8.56°, (022) to 12.09°, (113) to 14.18°, (222) to 14.79°, (004) to 17.11°, (133) to 18.56°, and (024) to 19.12° [44]. For comparison, a typical UiO-66-NH_2_ diffractogram can be found in the work by Vahidi et al. [45].

One can clearly see the differences between membranes fabricated by the one-pot method and those synthesized by the LPE–LBL procedure. The membranes from one-pot synthesis are characterized by two major peaks, while the U-LBL membrane exhibit only one wide peak in the small-angle range of the diffractogram. This difference is mainly due to the limited SURMOF thickness (ca. 100 nm) in the LBE-LBL process and a much thicker layer in the one-pot synthesis (in the range of μm) constituted by a bulk layer of crystals.

**Figure 4:**
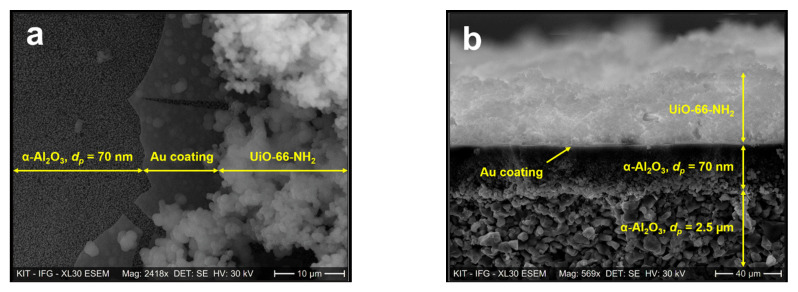
SEM images of (**a**) the top surface and (**b**) the cross-section of the U-LBL membrane.

Scanning electron microscopy imaging was conducted to analyze the topography of U-LBL and U-OP membranes. Figure 4a and b display SEM images of the top surface and cross-section of the U-LBL sample, respectively.

The image of the top surface, Figure 4a, presents three different layers in the UiO-66-NH_2_ membrane synthesized by LPE–LBL process on the Au-coated α-Al_2_O_3_ substrate. The uppermost granular layer, which is bright in the SEM image, is a sparse film of UiO-66-NH_2_. Beneath is the thin gold layer with its increased brightness in the cross-section image of Figure 4b due to a rise in the number of backscattered electrons interacting with the gold atoms. It is worth noting that, despite covering the whole substrate surface, the gold coating does not diffuse across its bulk. The α-Al_2_O_3_ support is composed of two different strata with pores having an average diameter of 70 nm in the upper part and pores with an average diameter of 2.5 μm in the lower layer. The SURMOF thickness cannot be determined precisely (i) due to the high roughness of the SURMOF surface and (ii) because the cross-section view is not exactly perpendicular to the cross-section plane (the top surface is, in fact, visible in Figure 4b).

**Figure 5:**
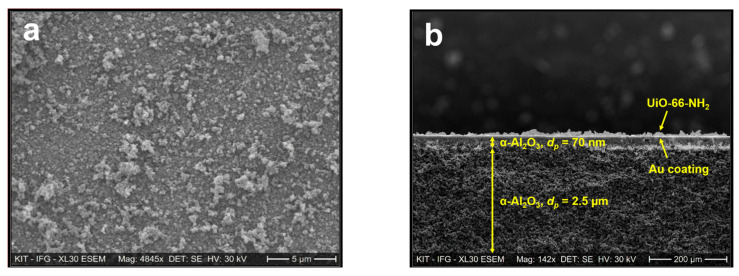
SEM images of (**a**) the top surface and (**b**) the cross-section of the membrane U-OP.

Figure 5a and 5b display a top view and a cross-section SEM image of the UiO-66-NH_2_ membrane grown via one-pot synthesis on an Au-coated α-Al_2_O_3_ substrate, respectively. One can see that the MOF distribution is more heterogeneous with U-OP compared to U-LBL synthesis. This is mainly due to the fact that the LPE–LBL method generates continuous and highly oriented frameworks, while one-pot-synthesized MOF crystals are randomly deposited on the support. As a result, multiple uncovered spots remain on the surface of U-OP. The MOF thickness is around 7.5 µm.

**Figure 6:**
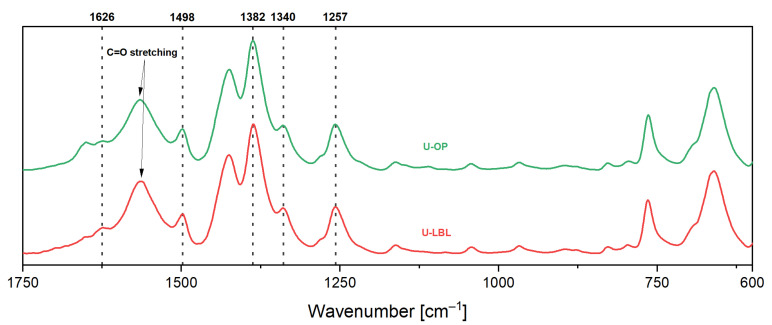
Normalized FTIR spectra of the UiO-66-NH_2_ powder of samples U-LBL and U-OP.

Figure 6 shows the FTIR spectra of the UiO-66-NH_2_ powder coming from the synthesis of membranes U-LBL and U-OP. The spectra were normalized to the highest peak intensity, which corresponds to the C–N stretching. They possess the characteristic infrared absorption bands of UiO-66-NH_2_, thus confirming the chemical composition of the MOF. The peak at 1626 cm^−1^ represents the typical N–H bending (scissoring). The presence of the C=C group is confirmed by the band at 1498 cm^−1^. The C=O stretching and the C–N stretching vibrations are observed at 1600–1503 and 1382 cm^−1^, respectively The bands at 1340 and 1257 cm^−1^ correspond respectively to the asymmetric and symmetric C–N stretching of the aromatic amines of the MOF structure [33,34,41,46,47,48].

### 3.2. Permeation Tests

The values for permeance *P* (mol·s^−1^·m^−2^·Pa^−1^) were computed using the following equation:

(1) $$P=\frac{n_{i}}{A\cdot \Delta p_{i}}$$

where *n_i_* (mol⋅s^−1^) is the permeate molar flow rate of species *i*, and *A* (m^2^) is the effective adsorption area of the membrane. Δ*p* (Pa) is the partial pressure across the membrane, i.e., between the permeate and retentate sides [49].

The permeation test was performed to identify the potential presence of cracks or large defects in the framework, which would cause a loss of selectivity and compromise the MOF capability of gas separation [39,50]. Indeed, the generation, reproducibility, and scalability of defect-free SURMOF-based membranes remain a challenge [44,50]. Different mass transport mechanisms arise in porous membranes depending on the pore size. Permeance dependency on pressure can be a helpful parameter to identify the dominant transport mechanism and consequently the average pore size of the sample.

**Figure 7:**
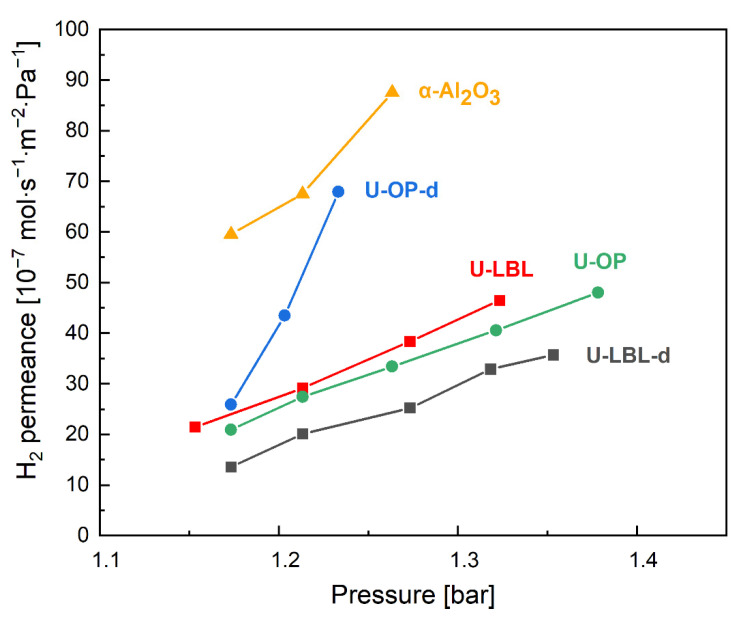
H_2_ permeance of the membranes from permeation test results conducted with increasing feed absolute pressure at room temperature.

Figure 7 displays the hydrogen permeances of U-LBL, U-OP, U-LBL-d, U-OP-d, and bare α-Al_2_O_3_ measured at room temperature with increasing absolute pressure. The permeance trends of U-LBL, U-LBL-d, and α-Al_2_O_3_ have been extracted from the article by Hashem et al. [35] and included in this graph for better comparability. As can be seen, U-OP-d exhibits a considerable permeance rise as pressure increases, which can be correlated with severe cracks on its surface. As a consequence, this sample was discarded in the gas separation experiments. Since the two LPE–LBL membranes have similar permeation trends, U-LBL was chosen as it shares the same Au-coated α-Al_2_O_3_ support of U-OP. As a result, the substrate can be removed as a factor impacting the separation results, allowing for a better understanding of the effect of the two synthesis procedures on the membrane performance.

The permeances at around 1.16 bar for U-OP and U-LBL are equal to 20.94·10^−7^ and 21.44·10^−7^ mol·s^−1^⋅m^−2^·Pa^−1^, respectively. These values are within the range of 25·10^−7^ mol·s^−1^·m^−2^·Pa^−1^ published by Jia et al. for this type of material [28]. One can note that the pressure dependency of permeance of U-LBL and U-OP samples is significantly reduced compared to bare α-Al_2_O_3_ support. However, a pressure dependency is observed for all samples; such behavior is unexpected due to the narrow diameter of triangular pore windows of 0.6 nm [51] and suggests the presence of microdefects and/or small cracks. The evolution of the permeance as a function of the pressure indicates a Knudsen diffusion as the predominant mass transport in U-LBL, U-OP, and U-LBL-d membranes, while for U-OP-d, it would be rather a viscous flow regime and, consequently, pores with a diameter larger than 50 nm [52].

### 3.3. Gas Separation Experiments

To assess the gas selectivity performance, the separation factor *SF* was used, as defined by the following equation:

(2) $$SF_{ij}=\frac{x_{i}^{P}x_{j}^{R}}{x_{j}^{P}x_{i}^{R}}$$

which evaluates the relative enrichment of the molar fraction *x* of species *i* over species *j* in the permeate stream (indicated with *P*) with respect to the composition in the retentate (*R*) [39]. In this work, hydrogen is referred to as component *i* and nitrogen as component *j*.

Table 1 summarizes the results of the gas separation experiments performed for the three different concentration ratios analyzed in this study.

As expected, the permeances for nitrogen are lower than for hydrogen based on molecular size and adsorption behavior with MOFs.

**Figure 8:**
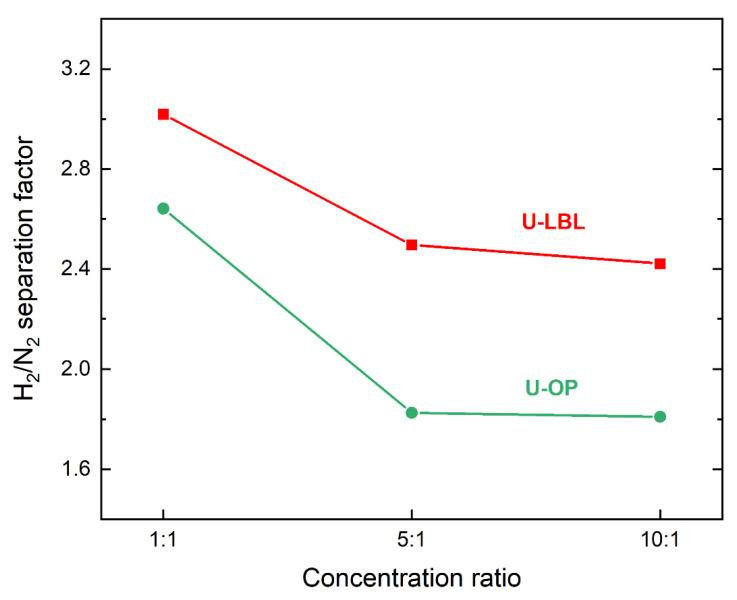
H_2_/N_2_ separation factors of the membranes in the three concentration ratios of 1:1, 5:1, and 10:1.

Figure 8 illustrates the H_2_/N_2_ separation factor trends with respect to the three different concentration ratios. The values decrease considerably, moving from an equimolar concentration to the 5:1 case compared to the change from 5:1 to 10:1. Indeed, from the equimolar to the 5:1 concentration ratio, a decrease equal to 17.2% for U-LBL and 30.7% for U-OP is observed, while the difference between the values of 1:1 to 10:1 concentration ratios is 19.9% for U-LBL and 31.4% for U-OP. Such a difference can be explained by the difference of N_2_ concentration with respect to the equimolar case, which is 33.3% and 18.2% for the 5:1 and 10:1 cases, respectively. These results imply that a decreased N_2_ concentration in the feed reduces the interactions between pore walls and N_2_ molecules, lowering the potential for effective adsorption. The non-ideal behavior of gas mixtures in diffusion processes through microporous membranes could also play a role.

The higher separation factor obtained with the U-LBL sample compared to the U-OP membrane is explained by the larger degree of surface continuity in this sample, as seen in the SEM images (Figure 4 and Figure 5). Thus, LPE–LBL method generating continuous and highly oriented frameworks allows achieving higher separation factors.

To the best of our knowledge, the separation factors reported in this study are the highest in the literature for single-phase UiO-66-NH_2_ membranes (i.e., without mixed-matrix membrane or other composite materials). Jia et al. reported that a UiO-66-NH_2_ membrane synthesized by solvothermal method on mixed cellulose ester filter support exhibited a mere 1.79 as H_2_/N_2_ ideal selectivity. The sample performance was then increased to 9.75 by adding 10 mg of graphene oxide nanosheets to 5 mg UiO-66-NH_2_ through vacuum filtration. Such an extraordinary increase in the gas separation values of MOF-based membranes is related to graphene oxide that has the potential to seal the non-selective gaps between MOF crystals [53,54] with the drawback of making the synthesis procedure more complex and costly.

In this study, it is worth mentioning that the performance of membranes was evaluated through the separation factor, as defined in Equation (2). In contrast, most research publications evaluate the separation performance of membranes based on ideal selectivity, calculated as the ratio of permeabilities of each pure species measured under similar conditions. This parameter oversimplifies the complicated diffusion process of gas mixtures in microporous membranes [55] by excluding any form of interaction between the species, such as competitive adsorption and diffusion [39,56]. As a result, numerous critical factors are ignored, [57] including molecular mass and diffusivity, particularly when light gases are involved. The ideal selectivity of a membrane is usually higher than its separation factor sometimes even more than twice its value and can be significantly misleading when evaluating separation performance [12]. Since the single-gas permeation of nitrogen was never measured, it was not possible to determine the ideal selectivity of the membranes produced in this study. However, a real selectivity can be computed as the ratio of the binary-gas permeances along the lines of the ideal selectivity, which is instead determined by unary permeation data. The computed values are 4.31 for U-LBL and 3.98 for U-OP, most likely lower than the ideal selectivity values [58].

## 4. Conclusions

The presence of nitrogen in a concentration above 300 ppm in the hydrogen fuel for vehicles is a major concern for several hydrogen refueling stations as the quality assurance required by the EU directives and standards is not ensured. Within this work, a fine-purification stage of hydrogen product is proposed using membranes based on MOFs and, in particular, UiO-66-NH_2_, which is one of the most promising ones due to its pore size as well as its thermal and chemical stability.

A UiO-66-NH_2_ SURMOF membrane grown by liquid-phase epitaxial layer-by-layer synthesis and a UiO-66-NH_2_ membrane prepared by the one-pot method were investigated for hydrogen purification, resulting in selective separation for hydrogen over nitrogen. The separation factors of the SURMOF and MOF membranes are equal to 3.02 and 2.64, respectively, in the equimolar case, but they drop as the hydrogen-to-nitrogen concentration ratio increases. The performances of the samples reported in this study are the highest compared to values reported in the literature for purifying hydrogen from nitrogen using single-phase UiO-66-NH_2_ membranes.

Moreover, it has been shown that the LPE–LBL method grows more selective MOF membranes compared to the solvothermal procedure. Indeed, the separation factor of the LPE–LBL sample in the equimolar case was 14.4% higher than the one-pot membrane. Since U-LBL and U-OP were characterized by the same MOF and support type, such a difference has been related to the high orientation and continuity of SURMOFs obtained from the LPE–LBL method.

The reported values are promising, and the next steps will aim at further improvement in the synthesis procedure in order to achieve separation factors close to the one requested for commercial applications.

## Figures and Tables

**Table 1:** H_2_/N_2_ separation factor and H_2_ and N_2_ permeance values of the two membranes tested in gas separation experiments.

Concentration Ratio	H_2_/N_2_ Separation Factor		H_2_ Permeance (10^−7^ mol⋅s^−1^⋅m^−2^⋅Pa^−1^)		N_2_ Permeance (10^−7^ mol⋅s^−1^⋅m^−2^⋅Pa^−1^)	
	U-LBL	U-OP	U-LBL	U-OP	U-LBL	U-OP
1:1	3.02	2.64	119.5	196.9	27.7	49.4
5:1	2.50	1.83	162.0	180.2	45.5	72.5
10:1	2.42	1.81	157.5	177.7	45.9	72.6

## Data Availability

Not applicable.
